# Large outbreak of Crimean-Congo haemorrhagic fever in Iraq, 2022

**DOI:** 10.1016/j.ijregi.2023.01.007

**Published:** 2023-01-18

**Authors:** Riyadh Abdulameer Alhilfi, Hanan Abdulghafoor Khaleel, Baghdad Muayad Raheem, Sinan Ghazi Mahdi, Celine Tabche, Salman Rawaf

**Affiliations:** a Directorate of Public Health, Ministry of Health, Baghdad, Iraq; b Communicable Diseases Control Centre, Directorate of Public Health, Ministry of Health, Baghdad, Iraq; c WHO Collaborating Centre for Public Health Education and Training, Department of Primary Care and Public Health, Imperial College London, London, UK

**Keywords:** Crimean-Congo Haemorrhagic Fever, CCHF, Outbreak, Haemorrhagic Fever, Iraq, EMR

## Abstract

**Background:**

Crimean-Congo haemorrhagic fever (CCHF) is reported sporadically in Iraq. The lack of preventive veterinary activities during 2 years of the coronavirus disease 2019 pandemic (2020 and 2021) led to the largest CCHF outbreak in Iraq since 1979.

**Objective:**

To describe the epidemiological characteristics of CCHF cases that occurred during the first half of 2022 in Iraq in terms of age, sex, residence, history of contact with another case, and history of contact with animals.

**Materials and methods:**

This descriptive study included laboratory-confirmed cases of CCHF between 1^st^ January 2022 and 26^th^ June 2022. Frequencies and percentages were used to describe the demographic and epidemiologic criteria of the cases. The epidemic curve of the cases was used to describe the timing and duration of the outbreak.

**Results:**

In total, there were 219 confirmed cases of CCHF from 1^st^ January 2022 to 26^th^ June 2022. The first confirmed case was reported in March 2022, and cases continued to occur through June 2022. The median age of the cases was 34.5 years. The majority of cases were male (*n*=130, 59.4%), had an unspecified job (*n*=126, 57.5%) and lived in southern Iraq (*n*=142, 64.8%). The first case was reported in week 10 of 2022. Case numbers peaked in week 24 (30 cases were reported), and subsequently declined in week 25 (24 cases were reported). The case fatality rate was 16.4%.

**Conclusion and recommendations:**

The CCHF outbreak in 2022 was the largest in Iraq since the disease was first reported four decades ago. Identification of CCHF strains in Iraq is recommended, together with exploration of the knowledge, attitudes and practices of high-risk groups for CCHF, and a national survey of CCHF vectors in Iraq.

## Highlights

• The outbreak of Crimean-Congo haemorrhagic fever (CCHF) in 2022 was the largest outbreak in Iraq in decades.
• A decline in preventive activities during the coronavirus disease 2019 pandemic resulted in a surge of cases.
• Despite the public health measures, there is a surge in the occurrence of CCHF worldwide.
• This increase is due to behavioural, environmental and ecologic factors.

## Introduction

Crimean-Congo haemorrhagic fever (CCHF) represents a major challenge to public health due to its burden on social and economic well-being, as well as its effects on the health of individuals [1]. Despite the presence of public health measures to control and prevent the spread of CCHF, there has been an increase in its occurrence worldwide, including in the Eastern Mediterranean region, over the last decade due to the nature of the disease, human behaviour, environmental and ecological factors, and improvements in diagnostic methods [1], [2], [3], [4].

CCHF is an acute tick-borne zoonotic disease caused by the Crimea-Congo virus of the *Nairoviridae* family [5]. CCHF is asymptomatic in 60–80% of cases, and the remaining 20–40% of cases usually suffer from initial fever, headache and malaise followed by gastrointestinal symptoms; severe cases can experience bleeding, shock and multi-organ system failure [6,7]. The geographical distribution of CCHF overlaps with the distribution of the hard tick vector – mainly *Hyalomma marginatum –* that is, Africa, Asia and Europe [8,9]. Turkey and Iran, which lie to the north and east of Iraq, are endemic for CCHF [10,11], and there have been reports of recent outbreaks and an increased number of cases [8,[12], [13], [14]]. A seroepidemiologic survey of CCHF in cattle, camels and sheep in 2016 found prevalence rates of 20–30% in Iraq, Iran and Turkey. In comparison, the prevalence of CCHF in goats was higher in all three countries (50%) [15].

Since the first report of CCHF in Iraq in 1979 [16], there have been sporadic outbreaks of disease interspersed with periods of no registered cases. Between 2007 to 2020, no cases were reported in 4 years (2008, 2014, 2016 and 2017), and the highest number of cases in any given year was 10 (in 2020). According to data from the Ministry of Health, Iraq, there has been a surge in cases over the last 2 years, with 19 laboratory-confirmed cases in 2021 and 108 laboratory-confirmed cases during the first half of 2022.

The main aim of this study was to describe the epidemiological criteria of patients with CCHF that occurred during the first half of 2022 in Iraq in terms of age, sex, governorate of residence, history of contact with another case, and history of contact with animals. The secondary aim was to identify potential areas for further research that may help to clarify the epidemiologic characteristics of CCHF in Iraq.

## Materials and methods

### Study design and population

This was a descriptive case series study of confirmed cases of CCHF in Iraq in 2022.

### Data source

This study used data on immediately notifiable diseases submitted routinely to the Surveillance Section, Communicable Disease Control Centre. All 20 health departments across Iraq are required to notify the Surveillance Section immediately regarding any suspected cases of CCHF, and should send all information within 72 h. The data were provided under the supervision of the Ministry of Health, Iraq, with ethical approval granted.

### Time

**Fig. 1:**
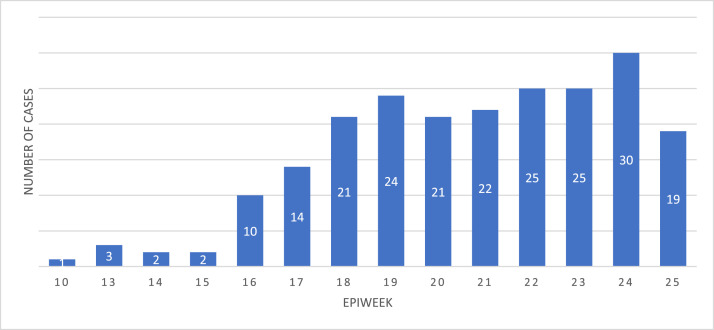
Weekly epidemic curve of confirmed cases of Crimean-Congo haemorrhagic fever cases in Iraq, 2022.

All data from cases of CCHF confirmed by reverse transcription polymerase chain reaction between 1^st^ January 2022 and 26^th^ June 2022 were included in the analysis (Fig. 1).

### Exposure variables

Variables on the immediate notification form include patient's name, age, sex, governorate of residence, occupation, history of contact with another case, history of contact with animals, patient outcome (recovery, death), date of notification, and date of outcome. Governorates in southern Iraq were highlighted and included Basra, Maysan, Muthanna, Diwaniya and Dhi Qar; the total population of these governorates was approximately 9.1 million (Table 1).

**Table 1:** Population of Iraq by governate, 2022.

Governorate	Population
Anbar	1,963,346
Babylon	2,288,456
Baghdad-Karkh	3,815,810
Baghdad-Resafa	5,190,191
Basrah	3,223,158
Dhi-Qar	2,321,851
Dahuk	1,432,369
Diwaniya	1,430,714
Diyala	1,814,368
Erbil	2,055,448
Kerbala	1,350,577
Kirkuk	1,770,765
Missan/Maysan	1,233,053
Muthanna	902,480
Najaf	1,630,807
Ninewa	4,133,536
Salah Al-Din	1,767,837
Sulaymaniya	2,396,206
Wassit	1,527,911
**Total**	42,248,883

### Analysis

**Fig. 2:**
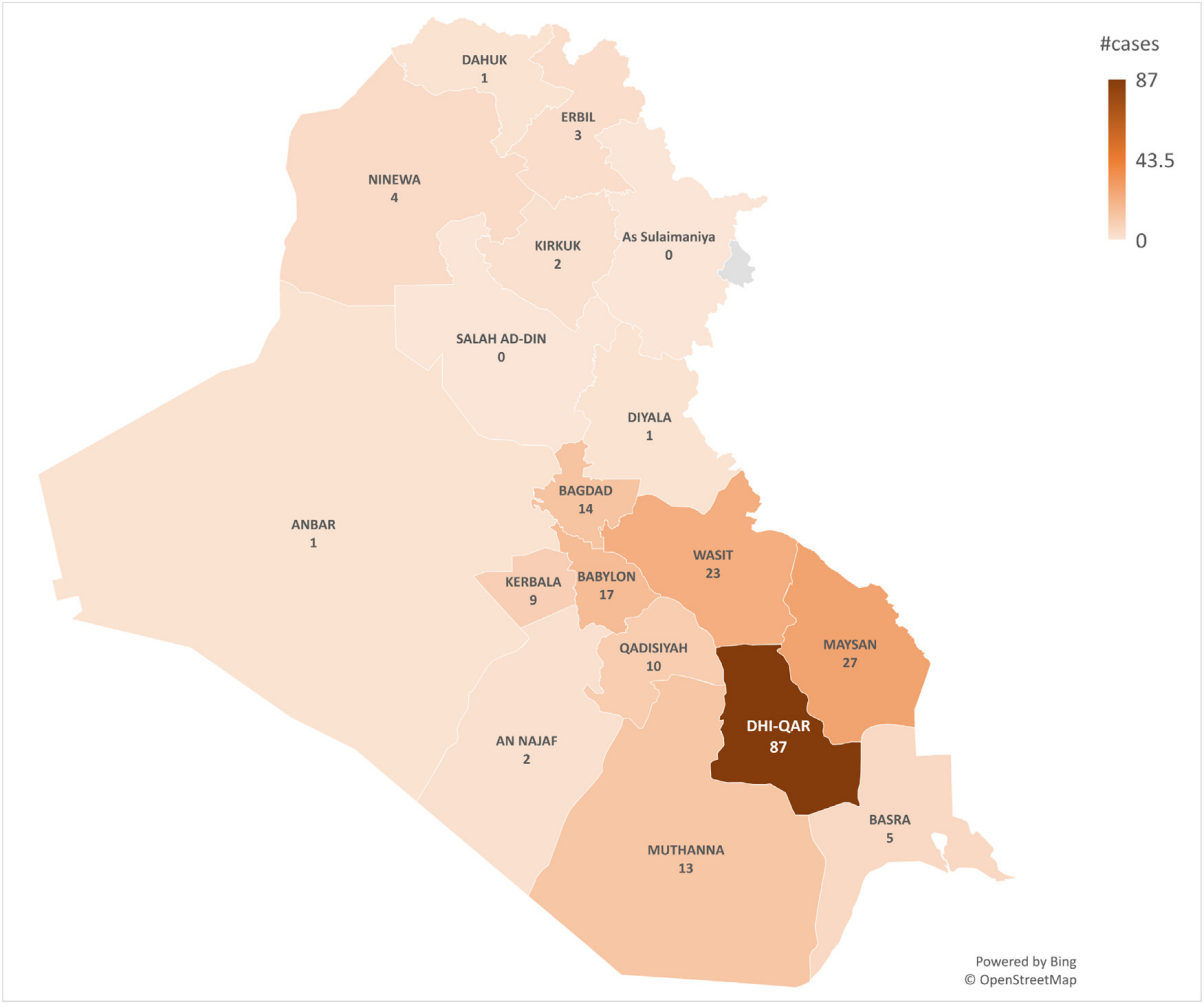
Distribution of laboratory-confirmed cases of Crimean-Congo haemorrhagic fever in Iraq, 2022.

Frequencies and percentages were used to describe the confirmed cases by age group, sex, governorate of residence, occupation, history of contact with another case, history of contact with animals, and patient outcome. Confirmed cases were mapped by governorate to identify clusters (Fig. 2). Data analysis was undertaken using Excel 2019.

## Results

In total, there were 219 confirmed cases of CCHF from 1^st^ January 2022 to 26^th^ June 2022 (Fig. 1). Most cases were aged 11–40 years (*n*=143, 65.3%), male (*n*=130, 59.4%), had an unspecified job (*n*=126, 57.5%) and lived in southern Iraq (*n*=142, 64.8%) (Table 2). The median age was 34.5 years (range 0.8–99 years). The first case was reported in week 10 of 2022. Case numbers peaked in week 24 (30 cases), and subsequently declined in week 25 (24 cases). The case fatality rate was 16.4% (Table 2).

**Table 2:** Epidemiological characteristics of positive cases of Crimean-Congo haemorrhagic fever in Iraq, 2022.

	*n* (%)	95% CI
**Total cases**	219 (100.0%)	
Median age (years) (range)	34.5 (0.8–99)	32.1–36.9
**Age groups**		
<1–10	3 (1.4%)	0.3–4.0%
11–20	45 (20.6%)	14.5–25.5%
21–30	48 (21.9%)	16.1–27.5%
31–40	50 (22.8%)	18.2–29.9%
41–50	41 (18.7%)	14.0–24.9%
51–60	17 (7.8%)	4.6–12.3%
61–70	10 (4.6%)	2.5–8.9%
71–80	5) 2.3%(	0.5–4.6%
**Sex**		
Male	130 (59.4%)	51.7–65.3%
Female	89 (40.6%)	34.7–48.3%
**Occupation**		
Housewife	29 (13.2%)	9.0–18.4%
Butcher	12 (5.4%)	2.8–9.3%
Military	5 (2.3%)	0.7–5.2%
Employed	9 (4.1%)	1.9–7.7%
Health care (farmer)	1 (0.4%)	0.01–2.5%
Students	23 (10.5%)	6.7–15.3%
Shepherd	14 (6.4%)	3.5–10.5%
Other	126 (57.5%)	50.6–64.2%
**Governorate of residence**		
Anbar	1 (0.5%)	0.01–2.6%
Babylon	17 (7.8%)	5.0–12.9%
Baghdad	14 (6.4%)	2.9–13.6%
Basra	5 (2.3%)	0.7–5.3%
Dhi-Qar	87 (39.7%)	33.6–47.1%
Diwaniya	10 (4.6%)	1.9–7.7%
Diyala	1 (0.5%)	0.01–2.6%
Duhok	1 (0.5%)	0.01–2.6%
Erbil	3 (1.4%)	0.3–4.0%
Kerbala	9 (4.1%)	1.6–7.1%
Kirkuk	2 (0.9%)	0.1–3.3%
Maysan	27 (12.3%)	7.6–16.6%
Muthanna	13 (5.9%)	3.2–10.1%
Najaf	2 (0.9%)	0.1–3.3%
Nineveh	4 (1.8%)	0.1–3.3%
Wasit	23 (10.5%)	6.8–15.5%
Shaded cells represent governorates in southern Iraq		
History of contact with another patient (yes) Missing/no (38)	4 (1.5%)	0.3––4.0%
History of contact with animals (yes) Missing/no (38)	59 (27.3%)	21.4–33.7%
Deceased	36 (16.4%)	11.6–22.0%

## Discussion

This study provides an epidemiologic description of the 2022 CCHF outbreak in Iraq that started in April 2022. The increased number of cases was reported in the infectious diseases surveillance update in *The Lancet* in July 2022 [17]. Most confirmed cases were male and living in southern Iraq, and the median age was 34.5 years. The case fatality rate was 16.4% (Table 2). The first confirmed case was detected in March 2022, and the outbreak is still ongoing through June 2022, typical of the seasonality of CCHF described elsewhere. A study of the seasonality of CCHF suggests that another wave could occur around August–October 2022 [18]. Intensive public health measures on the veterinary side, such as use of repellent and protective clothing, should be made as a response to the first wave in southern Iraq, and these should prevent, or at least reduce the severity of, a second wave.

The increase in the occurrence of CCHF in Iraq in 2022 can be explained by the increase in hard tick infestations of animals and farms. This increase may have occurred due to the absence of insect control activities in 2020 and 2021 during the coronavirus disease 2019 (COVID-19) pandemic. In addition, there is a lack of awareness about CCHF and its mode of transmission among butchers, farmers and the community. This is an important factor in the increasing infection rate, because humans are prone to getting the infection after a history of tick bites or animal handling. CCHF is most common in rural areas where wild and domestic animals serve as hosts to the tick vector, developing viraemia that aids maintenance and spread of the virus in nature [19]. The only hard tick species detected in Iraq as a vector for CCHF is *H. marginatum*. However, evidence suggests that 28 other species across seven genera could transmit the virus, such as *Hyalomma, Rhipicephalus, Boophilus, Ambylomma, Haemaphysalis* and *Ixodes* [20]. However, no studies have reported the detection of these species in Iraq.

This study describes the largest CCHF outbreak in Iraq since 1979. Limitations of this study include the lack of detailed medical histories and laboratory results for the cases, which restricts comparison between cases that recovered and cases that died. This comparison would provide insight into important prognostic factors that increase the chance of recovery. In addition, there was no information about CCHF strains of the confirmed cases, which could have provided further insight into the prognostic factors of the patients and the genetic variability of CCHF in Iraq. The fact that the majority of cases occurred in southern Iraq, and mainly in governorates bordering Iran, may be due to the illegal trading of animals across the borders. There is a need to control illegal trading activities in order to limit the spread of zoonotic diseases, including CCHF.

## Conclusion and recommendations

The CCHF outbreak in 2022 was the largest outbreak in Iraq in decades. The absence of preventive and control activities during the COVID-19 pandemic played an important role in the surge of cases in 2022. Identification of CCHF strains in Iraq is recommended, together with exploration of the knowledge, attitudes and practices of high-risk groups for CCHF, and a national survey of CCHF vectors in Iraq. Moreover, intensive preventive measures and enhanced human and vector surveillance activities should be carried out nationwide in preparation for a second wave that is expected to occur in August–October 2022.
